# Reproducibility of statistical data, academic publications and policy implications: Evidence from Ghana

**DOI:** 10.1016/j.dib.2018.04.008

**Published:** 2018-04-10

**Authors:** Samuel Kobina Annim

**Affiliations:** Department of Economics, University of Cape Coast, Cape Coast, Ghana

**Keywords:** Reproducibility, Sample Size, Comprehensiveness, Statistical Data and Ghana

## Abstract

This paper examines the accuracy, validity and presentation of statistical evidence and also assesses the implications of irreproducibility associated with variations in sample size for academic research work and policy-making. The 2012/13 Ghana Living Standards Survey (GLSS), 10 academic publications and the Free Senior High School policy in Ghana are used to address the objectives of the paper. The data show that about 20 per cent of the tables in the Main Report of the GLSS Six is irreproducible, 10 per cent of the tables have outcomes worth re-examining, and in terms of completeness in the presentation of statistical evidence, only 3 out of the 27 sampled tables report the sample size that was used. Again, nine out of the 10 academic publications use half of the original sample size, two-fifths of the publications do not report the sample size for the descriptive statistics, a couple of the papers show varying sample size between the descriptive statistics and the regression analysis.

**Specification Table**

**Table:** 

Subject Area	*Economics*
More Specific Subject Area	*Statistical Data and Public Policy*
Type of Data	*Table*
How Data was Acquired	*Survey*
Data Format	*Raw and Analysed*
Experimental Factors	*N/A*
Experimental Features	*N/A*
Data Source Location	*Ghana*
Data Accessibility	*Ghana Statistical Service*
Related Research Article	*N/A*

**Value of the Data**

The data provide comprehensive socio-economic information on Ghana at the regional and national levels. Among others, the data generally,

• Provide extensive database for national and regional planning. For instance, it provides a reliable impetus to conduct a comparative analysis on consumption as a proportion of household production.
• Provide information on trends of households’ consumption and expenditure at a greater level of disaggregation. Together with other surveys (GLSS1–5), the GLSS 6 provides the basis for comparing socio-economic and health issues on Ghana.
• Give a detailed investigation into the structure and distribution of wages and conditions of work of the country's labour force.
• Provide a wide-ranging standard data for use in the compilation of current statistics on average earnings, hours of work, and time rates of wages and salaries so as to indicate wage/salary differentials between branches of industry, geographic regions, occupations and the sexes

## 1 Data

The GLSS Six which was conducted in 2012–13 collects detailed information on Demographic Characteristics of Households, Education, Health, Employment, Migration and Tourism, Housing Conditions, Household Agriculture, Household Expenditure, Income and its Components and Access to Financial Services, Credit and Assets, Governance Peace and Security [1]. The data is hierarchical at the individual, household and community levels, thereby facilitating the analyses of both unit-specific and contextual issues that inform outturn of behaviours and policies. Official reports emanating from the GLSS Six are: the Main Report; Poverty Profile in Ghana 2005–2013; Labour Force Report; Child Labour Report; Governance Peace and Security Report; Water Quality Testing Report; and the Community Report [1].

The GLSS Six data, sourced from the GSS website, has 102 instrument-section specific (not including aggregated) data files and the number of variables in each of these data files ranges between 8 and 136. The Main Report of the GLSS Six, which has in total 212 tables and figures (excluding tables and figures in the annex), is used as the reference document for examining the accuracy of data through an attempt to reproduce a sample of the tables and figures. These tables and figures have been captured in 11 out of the 13 chapters (that is excluding the introductory and concluding chapters) of the GLSS Six Main Report. Guided by a minimum of two and a maximum of three tables and figures for each chapter (based on a pre-determined target of engaging at least 10% of the total number of tables and figures), stratified and simple random sampling techniques were employed. Two or three tables were selected depending on whether the total number of tables and figures in the chapter was less than or greater than 20. Specifically, two tables were selected in instances where the total number of tables was less than or equal to 20, while three tables were selected in the case where the number of tables and figures was more than 20 (See Appendix A).

The second objective of the paper assesses the implications of irreproducibility of statistical evidence as a result of non-availability and use or presentation of a varying number of observations. Ten working papers and journal articles published in the last three years (2015–17) and employing the GLSS Six raw data and regression techniques are used for the assessment. A conscious effort was made to select five papers each for individual and household level regression analysis. The population of working papers and journal articles that satisfy the above criteria was obtained using the google scholar, researchgate and sciencedirect search engines. The search yielded 11 papers, but one of them did not have full citation and therefore was dropped.

### 1.1 Materials and Methods

To minimise the occurrence of differences in reported statistical evidence and to initiate the culture of reproducibility, the STATA syntax used for generating the tables in this paper has been provided in Appendix B.

Table 1 shows that 80 per cent of the tables in the GLSS Six Main Report are reproducible. For all the chapters of the GLSS Six Main Report that had tables (Chapters 2 to 12), at least one sampled table was reproducible, thereby reposing a degree of confidence in the reliability of the reported statistical evidence across thematic issues. In seven out of the eleven chapters all sampled tables were reproduced. These were the chapters on Migration, Housing, Agriculture, Expenditure, Finance, Non-farm Enterprise and Governance. The inability to reproduce all the tables engenders the need to investigate the source of the difference. However, the ability to investigate and identify the source of variation strictly depends on the comprehensiveness of information and skills. Thus, the source of variation may emanate either from the author's error, lack of appreciation of the data manipulation process and/or assumptions underlying the variable generation process or from an error by GSS.

**Table 1:** Ability to reproduce statistical evidence reported in GLSS Six.

**Tables**	**Title of Table**	**Reproducible? (Yes or No)**	**Average Difference**^a^
Table 2.2	Average age of household heads by locality and sex	Yes	< 1.0
Table 2.10	Household heads by religion and locality	No	≥ 1.0
Table 3.1	Population aged 15 years and older by educational attainment and sex	No	≥ 1.0
Table 3.8	Adult literacy rates by sex and locality (read and write in English)	Yes	< 1.0
Table 4.3	Percentage of persons who reported ill and consulted a health practitioner during the two weeks preceding interview by age group, locality and sex	Yes	< 1.0
Table 4.7	Average consultation fees and payments for medicines (GH¢) two weeks preceding the interview (excluding those who paid nothing) by locality and sex	No	≥ 1.0
Table 4.15a	Percentage distribution of women aged 15–49 years who used contraceptives by amount paid and age group	No	≥ 1.0
Table 5.2	Current activity status by age, locality and sex	No	≥ 1.0
Table 5.5	Type of work engaged in by the currently employed population aged 15 years and older by locality and sex	Yes	< 1.0
Table 5.14	Currently employed population 15 years and older with contracts, unions, tax deductions and employee benefits by sex	Yes	< 1.0
Table 6.2	Migration status by region (per cent)	Yes	< 1.0
Table 6.7	Migrants by reason for most recent migration and locality of current resident	Yes	< 1.0
Table 6.13a	Domestic visitors by main purpose of visit and sex (per cent)		
Table 7.1	Households by type of dwelling and locality (per cent)	Yes	< 1.0
Table 7.6	Main materials used for outer wall of dwellings by locality (per cent)	Yes	< 1.0
Table 8.2	Estimated number of households raising livestock, livestock numbers and estimated values, sales and purchases of livestock	Yes	< 1.0
Table 8.5b	Estimated number of households engaged in fish farming, estimated value of sales and purchases by locality		
Table 9.2	Main source of capital for non-farm enterprises by industrial classification and sex (%)	Yes	< 1.0
Table 9.5	Expenditure on input for non-farm enterprises by type of activity (average)	Yes	< 1.0
Table 10.4	Expenditure components, mean annual per capita and estimate of total annual expenditure	Yes	< 1.0
Table 10.16	Average annual expenditure of households by type of dwelling occupied and locality (GH¢)	Yes	< 1.0
Table 10.17	Average annual expenditure of households by occupancy status and locality (GH¢)	Yes	< 1.0
Table 11.2	Type of account being held in financial institutions by locality and sex of individual (per cent)	Yes	< 1.0
Table 11.3	Proportion of households with members holding an insurance policy by locality	Yes	< 1.0
Table 12.9	Reasons for not reporting incidence of sexual offence to the police by region	Yes	<1.0
Table 12.13	Frequency of the incidence of crime in community or workplace in the 12 months preceding the survey by region	Yes	<1.0
Table 12.22	Households’ view of the extent to which government takes their views into account before changing laws by region	Yes	<1.0

a This is the difference between values computed in this paper and those of GSS averaged across the correlates of the focus of the table.

Generally, it was observed that derived variables and means, compared to proportions, were relatively challenging to reproduce. This is expected, since in practice the ability to reproduce statistical evidence from an existing dataset is enhanced through the provision of comprehensive metadata and the availability of a syntax file – a practice yet to be adopted by most producers and users of Ghanaian data. The limitedness of comprehensive documentation, especially a methodological manual for the GLSS Six, may account for the irreproducibility of derived variables. Also, the handling of outliers and variables with legitimate zero responses may account for the challenges in reproducing tables that have continuous outcomes.

For an appreciation of the extent of variation between statistical evidence produced in this paper and that reported in the GLSS Six Main Report, each of the tables that were irreproducible have been presented for discussion. This provides an opportunity to discuss the effect on overall patterns that will guide the discussion and policy recommendations.

From Table 2, more than 50 per cent of the values generated are reproducible. The variations are observed with the computation of the proportions of the different types of Christian religious denominations. The variations in the values generated in this paper and evidence from GSS range from 1.4 percentage points (proportions of Catholics in Accra (GAMA)) to 26 percentage points (proportions of Catholics in Rural Savannah). Noticeably, these variations do not change the patterns of proportions of different religious denominations across the geographical areas. However, it is conjectured that the source of deviation is emanating from the use of different number of observations in computing the proportions for the broad religious denomination compared to the sub categories (breakdown for the Christianity). Thus, the computation of the sub-categories of Christianity uses the sub-sample of Christianity as the total and the other broad religious categories use the total number of households as the sample size. While this is understandable, the non-provision of the different sample sizes for a single table may generate erroneous interpretation and use of the disaggregated data for further analysis.

**Table 2:** Irreproducibility of Table 2.10 – Household heads by religion and locality.

**Religious Denomination**	**Accra (GAMA)**		**Other Urban**		**Rural Coastal**		**Rural Forest**		**Rural Savannah**		**Total**	
	**Author**	**GSS**	**Author**	**GSS**	**Author**	**GSS**	**Author**	**GSS**	**Author**	**GSS**	**Author**	**GSS**
	**%**		**%**		**%**		**%**		**%**		**%**	
Christian	85.6	85.6	74.7	74.7	70.6	71.0	79.5	79.5	40.0	40.0	73.0	73.0
Catholic	7.9	9.3	11.1	14.8	10.5	14.7	14.1	17.7	17.3	43.3	12.1	16.5
Protestant	19.9	22.2	19.0	25.4	14.9	21.4	22.3	28.0	5.3	13.2	17.9	24.6
Pentecostal/Charismatic	46.5	54.4	32.5	43.5	34.0	47.6	30.6	38.5	12.9	32.2	32.0	43.8
Other Christian	11.3	13.2	12.1	16.2	11.2	16.3	12.6	15.7	4.5	11.3	11.0	15.1
Islam	11.8	11.8	20.7	20.7	17.9	17.5	9.4	9.5	51.9	51.9	20.2	20.2
Traditional	0.0	0.0	0.0	0.0	0.0	0.0	0.0	0.0	0.0	0.0	0.0	0.0
No religion	2.4	2.4	4.6	4.6	11.5	11.6	10.9	10.9	8.1	8.1	6.7	6.7
Other	0.3	0.3	0.1	0.1	0.0	0.0	0.1	0.1	0.0	0.0	0.1	0.1
Total	100.0	100.0	100.0	100.0	100.0	100.0	100.0	100.0	100.0	100.0	100.0	100.0

The GSS statistical evidence on educational attainment of the Ghanaian population aged 15 years and older is at variance with the values obtained from the reproducibility exercise. The observed differences span from -24.9 to 29.9 (see Table 3). Across all the values in Table 3, none of the values was reproducible and the variations affected the observed patterns of the proportion of educational attainment across the various levels. Specifically, while the GSS reports that the majority (44.6%) of Ghanaians aged 15 years and older have less than MSLC/BECE, the reproducibility exercise in the paper shows that the majority (45.6%) have attained MSLC/BECE/Vocational.

**Table 3:** Irreproducibility of Table 3.1 – Population aged 15 years and older by educational attainment and sex.

**Level of Educational Attainment**	**Both Sexes (%)**		**Male (%)**		**Female (%)**	
	**Author**	**GSS**	**Author**	**GSS**	**Author**	**GSS**
Never been to school	15.7	19.7	12.8	14.6	18.6	24.3
Less than MSLC/BECE	16.9	44.6	14.6	44.5	19.2	44.7
MSLC/BECE/Vocational	45.6	20.9	47.0	22.8	44.2	19.3
Secondary/SSS/SHS and higher	21.8	14.7	25.6	18.0	17.9	11.7
Total	100.0	100.0	100.0	100.0	100.0	100.0

Two tables (Tables 4.7 and 4.15a) out of the three that were sampled for the chapter on health were irreproducible. Table 4.7, titled “Average consultation fees and payments for medicines (GH¢) two weeks preceding the interview (excluding those who paid nothing) by locality and sex”, provided an opportunity to reproduce means to enhance comparison with reproduction of proportions. From the means reported in Table 4.7, for the eight sub-categories of health expenditure, the reproduction of one of them (consultation) was exact, while the averages for all the others varied significantly and in different directions. Thus, the reproduction exercise yielded values that were either greater than or less than the values reported in the GLSS Six Main Report. Compared to the values reported in the GLSS Main report, some of the variations from the reproduction exercise recorded a difference of more than 200 per cent. The outcomes for total medical expenses in Other Urban, Rural Forest and Rural Savannah areas point to such instances. The extent of the variation also affected the patterns of health expenditure across the geographical areas. For instance, the fees for overall treatment, as reported by GSS, show that the average cost in GAMA is greater than Other Urban areas by GH¢20.32, while the calculations from the reproducibility exercise show that a reverse pattern is observed where Other Urban areas have a higher mean expenditure by a margin of GH¢35.53.

Further scrutiny of the raw data revealed that there were significant outliers across the health expenditure categories. The reproduction exercise did not adjust the computation of the means to accommodate the effects of the outliers because there is no documentation on how extreme values were treated in the production of the statistical evidence in the GLSS Six Main Report. Although the health expenditure variables are directly obtainable from the raw data (not derived variables), the variations are significant relative to the earlier tables that were not reproducible. This can also be attributed to the influence of extreme values. It is worth interrogating this matter as in some instances the effects of the outliers were really compelling.

Table 4.15a, titled “Percentage distribution of women aged 15–49 years who used contraceptives by amount paid and age group”, is the second irreproducible table for the chapter on health. In contrast to Table 4.7, only two out of seven payment categorisations were irreproducible. However, through another lens, the extent of variations in these two cases is also high, as in the case of Table 4.7. Indeed, the difference in some instances was in excess of 500 percentage points. For example, payment bracket GH¢1.00 and GH¢1.99 for age group 20–24 shows a percentage difference of 804 per cent between the author's computation and the statistical evidence reported by GSS. Although values in the tables are percentages, they are underscored by the treatment of a continuous variable (amount paid for contraceptives). This supports the earlier proposition that continuous and derived variables are sensitive to reproducibility of statistical evidence relative to proportions. (Table 5)

**Table 4:** Irreproducibility of Table 4.7 – Average consultation fees and payments for medicines (GH¢) two weeks preceding the interview (excluding those who paid nothing) by locality and sex.

**Medical Expenses**	**Locality**																	
	**Ghana**						**Accra (GAMA)**						**Other Urban**					
	**Both Sexes**		**Male**		**Female**		**Both Sexes**		**Male**		**Female**		**Both Sexes**		**Male**		**Female**	
	Author	GSS	Author	GSS	Author	GSS	Author	GSS	Author	GSS	Author	GSS	Author	GSS	Author	GSS	Author	GSS
Consultation	16.60	16.60	17.33	17.33	16.00	16.00	15.20	15.20	13.72	13.72	16.62	16.62	15.19	15.19	11.93	11.93	16.90	16.90
Medicine	14.35	34.32	14.83	37.38	13.95	32.00	27.93	65.22	25.35	61.80	29.81	67.47	15.52	19.29	16.99	29.88	14.45	11.98
Total medical expenses	53.82	88.03	76.01	132.38	34.11	49.49	53.36	40.97	62.77	103.04	46.30	0.00	88.89	53.34	141.55	60.32	36.58	48.11
Registration	7.06	5.05	7.37	6.30	6.81	4.02	10.91	7.60	12.99	9.25	9.50	5.99	7.18	4.50	6.82	5.65	7.41	3.89
Diagnosis	31.35	13.28	36.52	20.09	31.35	7.66	48.89	22.52	53.36	29.28	46.40	16.05	26.42	17.15	40.40	37.98	18.71	6.17
Drugs	21.35	33.84	21.28	37.22	21.35	31.05	36.03	44.61	33.90	46.83	37.53	42.49	19.46	42.65	20.13	50.54	19.03	38.49
Overall treatment fees	53.82	47.03	76.01	48.83	53.82	45.58	53.36	65.62	62.77	67.37	46.30	63.94	88.89	45.30	141.55	36.37	36.58	50.12
Transport	5.72	4.85	5.81	5.40	5.72	4.40	8.16	5.47	8.60	6.56	7.90	4.43	4.91	3.50	6.06	3.33	4.19	3.58

**Medical Expenses**	**Locality**																	
	**Rural Coastal**						**Rural Forest**						**Rural savannah**					
	**Both Sexes**		**Male**		**Female**		**Both Sexes**		**Male**		**Female**		**Both Sexes**		**Male**		**Female**	
	Author	GSS	Author	GSS	Author	GSS	Author	GSS	Author	GSS	Author	GSS	Author	GSS	Author	GSS	Author	GSS
Consultation	28.37	28.37	18.25	18.25	34.25	34.25	19.99	19.99	29.24	29.24	11.59	11.59	13.82	13.82	19.16	19.16	7.48	7.48
Medicine	15.53	24.42	20.30	0.00	12.19	24.42	11.20	15.20	12.28	11.74	10.23	19.47	7.61	50.64	7.48	53.38	7.73	47.96
Total medical expenses	26.52	52.67	26.54	85.60	26.51	26.21	35.68	147.88	42.74	197.62	28.07	103.54	33.75	124.00	50.17	188.92	20.73	41.59
Registration	4.85	2.88	6.69	4.91	3.31	1.70	4.64	2.97	4.46	4.12	4.80	1.93	5.93	2.77	6.18	2.65	5.58	2.90
Diagnosis	14.45	2.52	6.39	4.71	19.26	1.24	17.01	0.88	21.28	1.64	14.42	0.19	15.25	0.57	7.58	0.98	22.20	0.05
Drugs	16.47	11.25	15.12	16.61	17.48	8.14	18.98	19.54	21.47	27.13	17.23	12.65	13.68	14.79	10.83	14.79	16.70	14.79
Overall treatment fees	26.52	23.05	26.54	38.80	26.51	12.76	35.68	23.45	42.74	29.94	28.07	18.72	33.75	28.91	50.17	30.48	20.73	27.07
Transport	6.79	7.07	5.60	4.18	7.45	8.75	5.53	6.06	5.16	7.29	5.79	4.95	5.57	3.25	5.02	3.30	6.03	3.19

**Table 5:** Irreproducibility of Table 4.15a – Percentage distribution of women aged 15–49 years who used contraceptives by amount paid and age group.

**Amount Paid (GH¢)**	**15–19**		**20–24**		**25–29**		**30–34**		**35–39**		**40–44**		**45–49**		**Ghana**	
	**Author**	**GSS**	**Author**	**GSS**	**Author**	**GSS**	**Author**	**GSS**	**Author**	**GSS**	**Author**	**GSS**	**Author**	**GSS**	**Author**	**GSS**
No payment	6.3	6.3	7.7	7.7	10.3	10.3	8.9	8.9	6.3	6.3	8.8	8.8	12.8	12.8	8.6	8.6
Less than 1.00	18.7	44.7	8.7	28.7	7.3	21.4	4.7	21.5	7.9	26.2	5.0	19.9	6.6	26.8	7.7	25.3
1.00–1.99	29.6	3.5	22.6	2.5	19.5	5.3	24.4	7.6	23.8	5.5	16.8	1.9	25.7	5.6	22.4	4.7
2.00–2.99	21.8	21.8	16.9	16.9	16.4	16.4	17.4	17.4	16.3	16.3	17.7	17.7	14.7	14.7	17.1	17.1
3.00–3.99	4.9	4.9	11.3	11.3	8.5	8.5	11.6	11.6	5.1	5.1	4.4	4.4	5.0	5.0	8.4	8.4
4.00–4.99	1.4	1.4	2.7	2.7	2.9	2.9	3.9	3.9	2.9	2.9	2.0	2.0	0.3	0.3	2.7	2.7
5.00 and more	17.3	17.3	30.1	30.1	35.1	35.1	29.2	29.2	37.7	37.7	45.4	45.4	34.7	34.7	33.1	33.1
Total	100	100	100	100	100	100	100	100	100	100	100	100	100	100	100	100
Avg. Amnt. Paid (GH¢)	2.3	2.2	4.0	3.0	4.5	3.2	3.8	3.1	7.5	3.1	10.7	3.3	10.9	2.8	5.4	3.1

The fifth table captured as irreproducible was identified in the chapter on employment. The unemployment rate in Ghana has, over time, attracted the attention of all stakeholders because of the low and differential rates across different rate surveys. The [1] based on the GLSS Six indicates that the unemployment rate in Ghana is 5.2 per cent while the [2] based on the LFS presents 11.9 per cent. Against the backdrop that issues related to conceptualisation, definition, standardisation, international benchmarking and local context are not interrogated in this paper, the observation on unemployment in Ghana calls for a re-consideration of these issues. Compared to other tables that were irreproducible, Table 6 presents relatively marginal differences between values obtained from the reproducibility exercise and the GLSS Six Main Report. Major variations were observed for the unemployed in Ghana. All the variables of economically active and inactive, employed and unemployed are all derived variables and require some assumptions, hence the need to have comprehensive information to inform reproducibility.

**Table 6:** Irreproducibility of Table 5.2 – Current activity status by age, locality and sex.

**Age / Locality**	**Economically Active**												**Economically Inactive**					
	**Employed**						**Unemployed**											
	**Male**		**Female**		**Total**		**Male**		**Female**		**Total**		**Male**		**Female**		**Total**	
	Author	GSS	Author	GSS	Author	GSS	Author	GSS	Author	GSS	Author	GSS	Author	GSS	Author	GSS	Author	GSS
**5–14 years**																		
Urban	12.9	13.0	15.3	15.4	14.1	14.2	0.0	0.4	0.0	0.4	0.0	0.4	87.1	86.6	84.7	84.2	85.9	85.4
Rural	34.7	34.8	32.5	32.9	33.6	33.9	0.0	0.2	0.0	0.1	0.0	0.1	65.3	65.0	67.5	67.0	66.4	66.0
Total	24.7	24.8	24.2	24.5	24.5	24.6	0.0	0.3	0.0	0.3	0.0	0.3	75.3	74.9	75.8	75.3	75.5	75.1
																		
**15+ years**																		
Urban	72.5	73.3	66.2	67.2	69.1	69.9	0.0	2.4	0.0	2.6	0.0	2.5	27.5	24.4	33.8	30.2	30.9	27.6
Rural	82.5	83.7	78.6	79.9	80.5	81.7	0.0	0.8	0.0	0.9	0.0	0.9	17.5	15.5	21.4	19.2	19.5	17.4
Total	77.4	78.4	72.0	73.1	74.5	75.5	0.0	1.6	0.0	1.8	0.0	1.7	22.6	20.1	28.0	25.1	25.5	22.8

### 1.2 Validity

A re-examination of this paper's Table 2, which is titled “Irreproducibility of Table 2.10 – Household heads by religion and locality”, shows that the column percentages, with the exception of respondents who are Christians, should sum up to 100 per cent as reported in the GLSS Six Main Report. Columns 3, 5, 7, 9 and 11 present totals exceeding 100 per cent. Cognisant of the fact that as a result of approximation the row or column percentages may sometimes vary by plus or minus 1 percentage point, the extent of variation from the expected 100 per cent in Table 2 ranges between 13.6 and 29.1 percentage points.

Table 7 provides an opportunity to compare the statistical evidence on educational attainment as documented in the Main Report of the GLSS Six with the GLSS Five (2005–06), 2010 GPHC, 2014 GDHS and computed values from this paper. Conscious of differences in dates for the respective census or surveys, assumptions underlying the grouping of educational attainment and variations in the use of educational level, grade, attainment and completion, the reported statistical evidence of the proportion of Ghanaians with less than MSLC/BECE in the GLSS Six varies strikingly in comparison with the other reports. Also, comparing the GLSS Six and the other surveys and census, the examination reveals variations in the proportion of Ghanaians who have obtained MSLC/BECE/Vocational.

**Table 7:** Validation of statistical evidence on educational attainment reported in GLSS Six.

**Level of Educational Attainment**	**GLSS Six 2012–13**	**GLSS Five 2005–06**	**GPHC 2010**	**GDHS 2014**	**Author's computation of GLSS Six**
Never been to school	19.7	30.8	28.4	22.0	15.7
Less than MSLC/BECE	44.6	17.1	11.6	15.4	16.9
MSLC/BECE/Vocational	20.9	38.6	36.1	44.1	45.6
Secondary/SSS/SHS and higher	14.7	13.6	23.8	18.5	21.8
Total	100.0	100.0	100.0	100.0	100.0

The third mode of validation of statistical evidence uses Table 4, which is titled “Irreproducibility of Table 4.7 – Average consultation fees and payments for medicines (GH¢) two weeks preceding the interview (excluding those who paid nothing) by locality and sex”, to examine veracity of the values. In this context, validity is assessed based on comparability of the obtained values across the localities. Based on broad expenditure patterns, expenditure in the Greater Accra Metropolitan Area (GAMA) is expected to be higher than all other four localities. Also, expenditure levels in urban areas are expected to be higher than rural areas. See Chapter 10 of the 2014 GSS Main Report. Of the eight sub- categories of health expenditure in Table 4, three (consultation fees, total medical expenses and transport expenses) depict that the mean expenditure in rural areas is greater than that for GAMA. Prima facie, this is contrary to the expected pattern and therefore calls for the ability to reproduce comparability to previous and comprehensive information on the processes, assumptions and context. Conscious of the fact that an argument can be put forward to support circumstances under which expenditure in rural areas would be higher than that of urban settlement, the scope (three out of the eight sub-categories) and intensity (extent of variation) undoubtedly call for further re-examination. Mindful of the sparsely distributed nature of health facilities in rural areas, which might culminate in relatively higher costs of health-related transportation compared to urban settlements, it would be quite challenging to ascribe reasons to the observation that total medical expenses and cost of consultation is higher in rural areas. Also, the average total medical expenses for all rural areas (Coastal, Forest and Savannah) are higher than for GAMA, being 200 per cent in excess for Rural Forest and Rural Savannah compared to GAMA.

To further obtain validity of the statistical evidence provided in Table 4.7, an examination of the aggregates using the mean of means of the sub-expenditure health categories across the localities is undertaken. From an urban-rural point of view, the mean of means shows that expenditure in urban areas is 12 per cent higher than rural settlements (GH¢234.07 and GH¢208.98 respectively). However, from Table 10.7 of the same GLSS Six Main Report, it can be observed that mean annual household cash expenditure for urban areas is 34.13 per cent higher compared to rural areas (GH¢169 and GH¢126 respectively). In addition to the above, the observations in 2012–13 were at variance with the pattern of health expenditure across the localities for the 2005–06 wave of the GLSS. In 2005–06, health expenditure for all the three sub-categories was higher in GAMA and urban areas compared to the other localities in rural areas. These observations corroborate the need for reproducibility of statistical evidence prior to engaging findings for policy-making and further analysis.

### 1.3 Comprehensiveness

The GLSS Six has more than 230 data files, making it too many for further analysis. This, coupled with the non-availability of a methodological manual on how the data files can be combined and assumptions and procedures for generating derived variables, increases the likelihood of errors in using the data for further analysis. The GDHS is organized in a compact manner and has extensive documentation on the processes for combining data files, as well as detailed information on the assumptions and procedures for generating derived variables.

Related to the packaging is the style and comprehensiveness in the presentation of the statistical evidence. For both the style and comprehensiveness, emphasis is placed on only one issue – handling and presentation of the number of observations. For any summary statistics to have meaning for a user, the number and elements of the observations are the most important factors. To this end, it is important for all statistical outputs to include the number of observations and their characteristics (including but not limited to applications of weights, conditions for inclusion and exclusion and issues related to standardisation and normalisation). Almost all the tables and figures in the GLSS Six provide minimal information on these expected attributes. Indeed, only one out of every ten tables in the GLSS provides information on the number of observations. The risk of not providing detailed information on the number of observations is that a statistic can be reproduced with a different set of observations that has varying attributes. Table 8 offers an example of this occurrence. Also, the treatment of weights is not discussed. Type of weight and its distinction across different units of analysis are critical pieces of information that have to be disclosed to improve the chances of reproducibility.

**Table 8:** Observations from Table 5.14 – Currently employed population 15 years and older with contracts, unions, tax deductions and employee benefits by sex – showing varying samples with approximate proportions.

**Correlates**	**Sample 15 years and older**						**Entire Sample**					
	**Male**		**Female**		**Total**		**Male**		**Female**		**Total**	
	**No.**	**%**	**No.**	**%**	**No.**	**%**	**No.**	**%**	**No.**	**%**	**No.**	**%**
**Signed written contract with employer**												
Yes, written	1703	38.7	828	37.0	2531	38.1	1706	38.4	829	36.6	2535	37.8
Yes, oral/verbal	1671	38.0	855	38.3	2526	38.1	1686	37.9	869	38.4	2556	38.1
No	1027	23.3	552	24.7	1579	23.8	1053	23.7	566	25.0	1618	24.1
Total	4401	100.0	2235	100.0	6636	100.0	4445	100.0	2264	100.0	6709	100.0
**Trade union available at work place**												
Yes	1322	30.1	593	26.5	1915	28.9	1324	29.8	594	26.2	1917	28.6
No	3074	69.9	1644	73.5	4718	71.1	3116	70.2	1672	73.8	4788	71.4
Total	4395	100.0	2237	100.0	6632	100.0	4440	100.0	2266	100.0	6705	100.0
**Taxes already deducted from pay**												
Yes	2415	30.1	1733	22.0	4148	26.1	2421	30.0	1737	22.0	4158	26.0
No	5612	69.9	6127	78.0	11,739	73.9	5647	70.0	6170	78.0	11,816	74.0
Total	8027	100.0	7860	100.0	15,887	100.0	8067	100.0	7907	100.0	15,974	100.0
**Entitled to paid holidays**												
Yes	1797	40.8	833	37.3	2630	39.6	1802	40.6	835	36.8	2637	39.3
No	2603	59.2	1403	62.7	4006	60.4	2642	59.4	1431	63.2	4072	60.7
Total	4400	100.0	2237	100.0	6636	100.0	4444	100.0	2265	100.0	6709	100.0
**Entitled to paid sick leave or maternity leave**												
Yes, sick leave	1695	38.5	403	18.0	2098	31.6	1698	38.2	404	17.8	2102	31.3
Yes, maternity	260	5.9	221	9.9	481	7.3	261	5.9	221	9.8	482	7.2
Yes, both	194	4.4	434	19.4	627	9.5	194	4.4	434	19.2	628	9.4
No	2251	51.2	1179	52.7	3429	51.7	2292	51.6	1205	53.2	3497	52.1
Total	4400	100.0	2237	100.0	6636	100.0	4444	100.0	2265	100.0	6709	100.0
**Receive any retirement pension**												
Yes	1387	31.5	595	26.6	1982	29.9	1389	31.2	596	26.3	1985	29.6
No	3013	68.5	1642	73.4	4654	70.1	3055	68.8	1669	73.7	4725	70.4
Total	4400	100.0	2237	100.0	6636	100.0	4444	100.0	2265	100.0	6709	100.0
**Entitled to any social security**												
Yes	1344	30.5	588	26.3	1932	29.1	1346	30.3	588	26.0	1934	28.8
No	3056	69.5	1649	73.7	4705	70.9	3099	69.7	1678	74.0	4777	71.2
Total	4400	100.0	2237	100.0	6637	100.0	4445	100.0	2266	100.0	6711	100.0
**Entitled to subsidized medical care**												
Yes	932	21.2	365	16.3	1298	19.6	934	21.0	367	16.2	1301	19.4
No	3467	78.8	1871	83.7	5338	80.4	3510	79.0	1898	83.8	5408	80.6
Total	4400	100.0	2237	100.0	6636	100.0	4444	100.0	2265	100.0	6709	100.0

Table 5.14 of the GLSS Main Report, titled “Currently employed population 15 years and older with contract, unions, tax deductions and employee benefits by sex”, is used to demonstrate the relevance of presenting the number of observations used in generating statistical evidence. From Table 8 in this paper, the column captioned “Sample 15 Years and Older” exactly reproduces the statistical evidence documented in the GLSS Six Main Report. The column captioned “Entire Sample” ignores the age restriction (15 years and older) and generates the statistical evidence for all those who are working. Using an approximation condition of less than one per cent all the statistical evidence will be reproduced, but with a different sample specifically including children. It is worth noting that, based on the correlates presented in Table 8, children are not likely to benefit from issues related to contract, unionism, tax holidays, social security and pension schemes. Thus, approximate statistical evidence has been reproduced in an instance where the sample variance is 73 (6709 for the entire sample compared to 6636 for the sample with the age restriction – see the last row of Table 8). The implications of the observed variations in the sample size from Table 8 will surface as the data is further engaged for detailed analysis. This is so because, for instance, use of averages responds to outliers whereas cross tabulations do not. Such occurrence may lead to erroneous policies and/or counterintuitive results.

### 1.4 Comparability of sample sizes employed

The rest of the presentation responds to the second objective by providing a discussion on how variation in sample sizes influences the outcome of summary statistics, regression analysis and policy direction. Table 9 presents sample sizes used for descriptive statistics, regression analysis of the 10 working papers and journal articles and shows how these compare with the original sample as provided in the GLSS.

**Table 9:** Sample sizes used for academic publications and GLSS data.

**No.**	**Citation of paper**	**Primary unit of analysis**	**Sample – descriptive**	**Sample – regression**	**Proportion of GLSS sample**	**Information on data restriction**
1	Iddrisu, A. M., Danquah, M. and Quartey, P. (2017) Paying for education among households in Ghana: Is there any role for household resources and contextual effects*? International Journal of Development Issues 16*(2), 214–226.	Household	Not indicated	5270	0.31	–
2	Nunoo, J., Darfor, K. N., Koomson, I. and Arthur, A. (2016) Employment Security and Workers’ Moonlighting Behaviour in Ghana. AGDI Working Paper, No. WP/16/006	Individual	2965	2965	0.04	–
3	Molini, V., Pavelesku D. and Ranzani, M. *Should I Stay or Should I Go? Internal Migration and Household Welfare in Ghana (July 20, 2016)*. World Bank Policy Research Working Paper No. 7752.	Household	6253	6383	0.38	–
4	Amponsah, S. (2016). The Incidence of Health Shocks, Formal Health Insurance, and Informal Coping Mechanisms. *Perspectives on Global Development and Technology*, *15*(6), 665–695.	Household	16,772	16,772	1.0	–
5	Mahé, C. and Naudé, W. *Migration, occupation and education: Evidence from Ghana (2016).* UNU-MERIT working paper 2016, 018.	Individual	27,037	27,037/27,209	0.37/	Individuals aged 16–64 years
6	Baah-Boateng, W. (2015). Unemployment in Ghana: a cross sectional analysis from demand and supply perspectives. *African Journal of Economic and Management Studies*, *6*(4), 402–415.	Individual	Not indicated*	32,771	0.45	Unemployed individuals aged 15+ years with 30-day job search period
7	Afoakwah, C. and Dauda, F. (2016) Employment Status and educational attainment among disabled Ghanaians. WIDER Working Paper 2016/56	Individual	19,108	19,108/26,717	0.26/0.82	Children aged 4–15 years
8	Danquah, M. and Ohemeng, W. (2017). Unmasking the factors behind income inequalities in Ghana. *International Journal of Social Economics*, (just-accepted), 00–00	Household	Not indicated	Not indicated	–	–
9	Amuakwa-Mensah, F., Boakye-Yiadom, L. and Baah-Boateng, W. (2016). Effect of education on migration decisions in Ghana: a rural-urban perspective. *Journal of Economic Studies*, *43*(2), 336–356.	Individual	Not indicated*	18,452	0.25	Individuals aged 15–60+ years
10	Koomson, I. and Asongu, S. A. (2016). Relative Contribution of Child Labour to Household Farm and Non-Farm Income in Ghana: Simulation with Child's Education. *African Development Review*, *28*(1), 104–115.	Household	1506	1658/1506	0.02	Households with non-zero income

Table 9 shows that four out of the ten working papers and journal articles did not indicate the sample size for the descriptive statistics and one of these four papers failed to indicate the sample size for both the descriptive statistics and the regression analysis. For the six papers that reported sample size for both the descriptive statistics and the regression analysis the values were the same, with the exception of two cases. In these two cases, the sample size for the regression analysis was greater than that for the descriptive statistics. Based on the original sample size for the GLSS Six (16,772 households and 72,372 individuals), the last column of Table 9 computes the proportion of the sample that was used for the regression analysis of each of the ten working papers and journal articles.

### 1.5 Variations in locational patterns based on differences in sample sizes

Table 10, Table 11 provide a platform to assess the distribution of respondents across geographical areas that were critical to the sampling procedure of the original data. Regional representation was prioritised in the implementation of the GLSS and reflects population density in the respective regions of the country. Columns 2 and 3 of Table 10 present the urban and regional distribution of households and individual respondents in Ghana. Columns 4 and 5 of the same Table present the distribution of Ghanaians based on the samples used by the two papers. It is observed that the distribution of households across the urban and regional areas is the same (Columns 2 and 4). This is expected given the same sample size. Contrary to this observation is the variation in the patterns for columns 3 and 5, where the sample sizes are not the same. For example, the evidence from the GSS indicates that the third most populous region is the Eastern, but from the fifth column Northern is the third most populous region. The concern, as presented in Table 11, is accentuated in the case of Paper No. 3. The regional distributions for both sub-samples (migrant and non-migrant) point to the fact that Upper West is the most populous region and Greater Accra is one of the two least populous regions in Ghana. While admittedly, the authors provide detailed procedures and reasons for using a reduced sample of 6383 households instead of 16772, the observed variations present a concern for generalisability of the findings. The verification and assessment of the implications of using a reduced sample are worth discussing in academic publications.

**Table 10:** Distribution of respondents in Paper Nos. 4 and 5 of Table 9 and the GLSS Six.

**Geographical Location**	**GLSS Six (Household)**	**GLSS Six (Individual)**	**Paper No. 4 (Household)**	**Paper No. 5 (Individual)**
Urban	55.39	50.06	55.39	49.87
Western	9.18	9.28	9.18	8.46
Central	9.27	8.80	9.27	9.88
Greater Accra	18.95	16.28	18.95	14.15
Volta	7.97	8.72	7.97	8.11
Eastern	10.93	10.35	10.93	9.90
Ashanti	21.22	19.66	21.22	20.46
Brong Ahafo	9.31	10.01	9.31	9.04
Northern	7.45	9.98	7.45	11.28
Upper East	3.64	4.04	3.64	5.00
Upper West	2.09	2.87	2.09	3.71
**SAMPLE**	**16,772**	**72,372**	**16,772**	**27,209**

**Table 11:** Distribution of respondents in Paper No. 3 of Table 9 and the GLSS Six.

**Geographical Location**	**Proportions**			
	**Migrant**	**GLSS Six**	**Non-migrant**	**GLSS Six**
Urban	0.56	0.54	0.50	0.49
Western	0.05	0.10	0.08	0.08
Central	0.16	0.08	0.10	0.10
Greater Accra	0.05	0.21	0.12	0.13
Volta	0.13	0.09	0.08	0.08
Eastern	0.14	0.11	0.10	0.10
Ashanti	0.12	0.20	0.18	0.20
Brong Ahafo	0.09	0.10	0.07	0.10
Northern	0.12	0.07	0.16	0.12
Upper East	0.08	0.03	0.06	0.06
Upper West	0.72	0.02	0.70	0.04
**Sample**	**1101**	**10,535**	**5282**	**6195**

### 1.6 Policy implications of irreproducibility

The value associated with evidence-based policy-making has been well documented [3] leading to its advocacy in all circumstances including interventions that are undeniably needed. To this end, while the Free SHS policy in Ghana is undeniably a worthy cause, it is imperative to assess the role of data in supporting the policy direction and in tracking the outputs, outcomes and impact. As a precursor to the discussion on the implications of irreproducibility for policy-making and tracking, it is important to interrogate the type of data needed for a particular policy. For the purposes of this paper, it is assumed that education has been prioritised among other issues and the concern is the choice of investing in a particular educational level at a given point in time. The data required to reach a decision on a particular educational level will need to capture the mobility of pupils and students across different levels to determine the entry level that requires focused intervention. This will mean that panel data for a particular cohort of children will serve the purpose. Cross-sectional data, like the GLSS, will provide only a snapshot of information and is therefore not ideal for tracking performance over time. However, since there is no long-term national data designed to track the enrolment and graduation of a given cohort of pupils and students, the GLSS comes in handy. With this limitation in mind, it is imperative to ensure that the available cross-sectional data is devoid of other challenges such as non-comparability across different surveys and censuses and irreproducibility.

With reference to Table 7, the second and third columns reported variations in the patterns across the four levels of educational attainment, and these provide different leads on the level to be prioritised. The evidence reported in the GLSS Six lends itself to focusing on MSLC/BECE, since 44.6 per cent of Ghanaians are unable to attain this level. Thus, the evidence from the GLSS Six does not support the Free SHS policy. However, the GLSS Five indicates that about two-fifths of Ghanaians attain MSLC/BECE/Vocational and only 13.6 per cent complete Secondary/SSS/SHS or higher. Therefore, the evidence in the GLSS Five aligns with the rationale for the Free SHS. The statistical evidence reported in the GLSS Six on educational attainment is irreproducible. This engenders further concern, especially in the light of the fact that the calculations in this paper support the Free SHS. The experience of irreproducibility dampens confidence in the data and adversely affects the use of the data to inform policy. This further justifies the clarion call for the need to provide comprehensive documentation, including a methodological manual and syntax for generating statistical evidence, in the release of nationally conducted surveys such as the GLSS.

## Footnotes

Transparency document Transparency data associated with this article can be found in the online version at https://doi.org/10.1016/j.dib.2018.04.008.
