# Supplementary material for: Reproducibility of statistical data, academic publications and policy implications: Evidence from Ghana
Source: Data Brief. 2018 Apr 10;18:1298–312. doi: 10.1016/j.dib.2018.04.008 (PMC5996736; doi:10.1016/j.dib.2018.04.008)
Supplement: Supplementary file 1 — Supplementary material [file mmc1.docx]

**Conflict of Interest Form**

There is no conflict of interest.
