# Supplementary material for: Reproducibility of statistical data, academic publications and policy implications: Evidence from Ghana
Source: Data Brief. 2018 Apr 10;18:1298–312. doi: 10.1016/j.dib.2018.04.008 (PMC5996736; doi:10.1016/j.dib.2018.04.008)
Supplement: Supplementary file 2 — Supplementary material [file mmc2.docx]

*Appendix 1:* Table A1: Population and Sample of Tables and Figures in the GLSS Six Main Report

| **Chapters** | **Pop. (N)** | **Sample (n)** | **Sampled table** |
| --- | --- | --- | --- |
| Two - Demographic Characteristics | 12 | 2 | 2.2 and 2.10 |
| Three - Education | 12 | 2 | 3.1 and 3.8 |
| Four - Health | 28 | 3 | 4.3, 4.7 and 4.15a |
| Five - Employment | 23 | 3 | 5.2, 5.5 and 5.14 |
| Six - Migration and Tourism | 27 | 3 | 6.2, 6.7 and 6.13a |
| Seven - Housing | 16 | 2 | 7.1 and 7.6 |
| Eight - Agriculture | 20 | 2 | 8.2 and 8.5b |
| Nine - Non-Farm Enterprises | 7 | 2 | 9.2 and 9.5 |
| Ten - Expenditure, Income and Their  Components | 29 | 3 | 10.4, 10.16 and 10.17 |
| Eleven - Access to Financial and Insurance  Services, Credit and Assets | 15 | 2 | 11.2 and 11.3 |
| Twelve - Governance, Peace and Security | 23 | 3 | 12.9, 12.13 and 12.22 |
| Total (%) | **212** | **27(12.7%)** |  |

*Appendix 2:* STATA Syntax for generating tables

1. **Syntax for Sampled Tables in Chapter Two**

Setting up data

*/*Use Aggregated Files*/*

*use "AGGREGATES/GHA_2013_H.dta"*

*merge 1:1 HID using "AGGREGATES/00_GHA_BASICINFO.dta"*

Table 2.2

*recode RURURB (2=0 urban) (1=1 rural), gen(rural)*

*recode HHSEX (1=0 male) (0=1 female), gen(female)*

*table loc5 female [aw=WTA_S], contents(mean HHAGEY) by(rural) format(%9.1f) ///*

*row col con*

*/*Use disaggregated Files*/*

*use "PARTA/SEC1.dta", clear*

*merge m:1 HID using "AGGREGATES/00_GHA_BASICINFO.dta"*

*keep if s1q3==1*

*recode RURURB (2=0 urban) (1=1 rural), gen(rural)*

*table loc5 s1q2 [aw=WTA_S], contents(mean s1q5y) by(rural) format(%9.1f) row col con*

Table 2.10

*recode s1q10 (2 3 4 5=1 Christian) (1 6/9 =0 "Not Christian"), gen(christian)*

*tab s1q10, gen(rel)*

*renames rel* \ no_religion catholic protestant pent_char oth_chri islam ///*

*ahmadiya traditinal other*

*replace islam=1 if ahmadiya==1 & islam==0*

*tabout christian no_religion catholic protestant pent_char oth_chri ///*

*islam traditinal other loc5 [aw=WTA_S] using table_2.10.xls, c(col) layout(cb) replace*

1. **Syntax for Sampled Tables in Chapter Three**

Setting up data

*use "PARTA/SEC1.dta"*

*merge 1:1 HID PID using "PARTA/SEC2a.dta"*

*keep if _merge==3*

*drop _merge*

*merge 1:1 HID PID using "PARTA/SEC2b.dta"*

*drop _merge*

*merge 1:1 HID PID using "PARTA/SEC2c.dta"*

*drop _merge*

*merge m:1 HID using "AGGREGATES/00_GHA_BASICINFO.dta"*

Table 3.1

*recode s2aq3 (0 1 =1 "Never been to school") (2=2 "Less than MSLC/BECE") ///*

*(3 4 7 =3 "MSLC/BECE/Vocational") (5 6 8/11=4 "Secondary/SSS/SHS and higher"), gen(educ_level)*

*tab educ_level s1q2 if s1q5y>=15 [aw=WTA_S], col*

Table 3.8

*des s2cq1 s2cq3*

*gen literacy=1 if s2cq1==1 & s2cq3==1 & s1q5y>14*

*replace literacy=1 if s2cq1==3 & s2cq3==3 & s1q5y>14*

*replace literacy=0 if s2cq1!=. & literacy!=1 & s1q5y>14*

*replace literacy=100 if literacy==1*

*table s1q2 loc5 loc2 [aw=WTA_S], c(mean literacy) ///*

*format(%9.1f) row col con sc*

1. **Syntax for Sampled Tables in Chapter Four**

Setting up data

*use "PARTA/SEC1.dta"*

*merge 1:1 HID PID using "PARTA/SEC3a.dta"*

*drop _merge*

*merge 1:1 HID PID using "PARTA/SEC3b.dta"*

*drop _merge*

*merge 1:1 HID PID using "PARTA/SEC3c.dta"*

*drop _merge*

*merge 1:1 HID PID using "PARTA/SEC3d.dta"*

*drop _merge*

*merge 1:1 HID PID using "PARTA/SEC3e.dta"*

*drop _merge*

*merge m:1 HID using "AGGREGATES/00_GHA_BASICINFO.dta"*

*drop _merge*

*merge m:1 HID using "AGGREGATES/POV_GH_2013.dta"*

*drop _merge*

*merge 1:1 HID PID using "PARTA/SEC2a.dta"*

Table 4.3

*rename s1q5y age*

*recode age (min/5=1 "0 - 5") (6/19=2 "6 - 19") (20/49=3 "20 - 49") ///*

*(50/max=4 "50+"), gen(age_cat)*

*recode s3aq1 (1=0 not_ill) (2 3 4 =1 ill), gen(illness)*

*recode s3aq5 (1=1 Yes) (2=0 No), gen(consult)*

*gen consult_1=consult*100*

*table age_cat s1q2 loc5 [aw=WTA_S] if illness==1, c(mean consult_1) ///*

*format(%9.1f) row col sc*

Table 4.7

*foreach x of varlist s3aq9-s3aq15 s3aq22 s3aq23 {*

*replace `x'=. if `x'==0*

*}*

*su s3aq9 s3aq10 s3aq11 s3aq12 s3aq13 s3aq14 s3aq15 s3aq22 s3aq23*

*foreach x of varlist s3aq9-s3aq15 s3aq22 s3aq23 {*

*replace `x'=. if `x'==99999.99*

*}*

*rename s3aq9 registration*

*rename s3aq10 consultation*

*rename s3aq11 diagnosis*

*rename s3aq12 amount_drugs*

*rename s3aq13 overall_treatment*

*rename s3aq15 transport*

*rename s3aq22 amount_medicine*

*rename s3aq23 total_medical*

*table loc5 s1q2 [aw=WTA_S] if illness==1, content(mean consultation ///*

*mean amount_medicine mean total_medical mean registration) format(%9.2f) row col sc*

*table loc5 s1q2 [aw=WTA_S] if illness==1, content(mean diagnosis mean ///*

*amount_drugs mean total_medical mean transport) format(%9.2f) row col sc*

Table 4.15a

*recode age (15/19=1 "15 - 19") (20/24=2 "20 - 24") (25/29=3 "25 - 29") ///*

*(30/34=4 "30 - 34") (35/39=5 "35 - 39") (40/44=6 "40 - 44") ///*

*(45/49=7 "45 - 49") if s1q2==2, gen(age_cat_w)*

*replace age_cat_w=. if age<15 | age>49*

*recode s3eq4 (0=1 "No payment") (0.00001/0.99999=2 "less than 1.00") ///*

*(0.999999/1.99999=3 "1.00-1.99") (1.9999999/2.99999=4 "2.00-2.99") ///*

*(2.9999999/3.99999=5 "3.00-3.99") (3.9999999/4.99999=6 "4.00-5.99") ///*

*(4.9999999/max=7 "5 and more"), gen(con_amt)*

*tab con_amt age_cat_w [aw=WTA_S] if s3eq3==1, col nofreq*

*tab age_cat_w [aw=WTA_S] if s3eq3==1, su(s3eq4)*

1. **Syntax for Sampled Tables in Chapter Five**

Setting up data

*use PARTA/SEC4d.dta, replace*

*merge 1:1 HID PID using "PARTA/SEC1.dta", gen(merge1)*

*merge 1:1 HID PID using PARTA/SEC4a.dta, gen(merge2)*

*merge 1:1 HID PID using PARTA/SEC4b.dta, gen(merge3)*

*merge 1:1 HID PID using PARTA/SEC4c.dta, gen(merge4)*

*merge 1:1 HID PID using PARTA/SEC4d.dta, gen(merge5)*

*merge 1:1 HID PID using PARTA/SEC4e.dta, gen(merge6)*

*merge 1:1 HID PID using PARTA/SEC4f.dta, gen(merge7)*

*merge 1:1 HID PID using PARTA/SEC4g.dta, gen(merge8)*

*merge 1:1 HID PID using PARTA/SEC4h.dta, gen(merge9)*

*merge 1:1 HID PID using PARTA/SEC4hs.dta, gen(merge10)*

*merge m:1 HID using AGGREGATES/00_GHA_BASICINFO.dta, gen(merge12)*

*rename s1q5y age*

*rename s1q2 sex*

TABLE 5.2

*gen ageca=.*

*replace ageca=1 if age>=5 & age<=14*

*replace ageca=2 if age>=15*

*recode ageca (1=1 "5-14") (2=2 "15+"), gen(agecategor)*

*gen employ=s4aq1==1 | s4aq2==1 | s4aq3==1*

*recode employ (0=0 "unemployed") (1=100 "employed"), gen(employed)*

*gen not_active=(employed==0)*

*replace not_active=0 if s4dq1<=2*

*replace not_active=1 if s4aq2==3*

*replace not_active=1 if s4dq10==1 | s4dq10==2 | s4dq10==8*

*recode not_active (0=0 "Active") (1=100 "Not Active"), gen(notactive)*

*tab notactive [aw=WTA_S]*

*gen unemployed=(employed==0 & not_active==0)*

*recode unemployed (0=0 No) (1=100 Yes), gen(unemploy)*

*table loc2 sex agecategor [aw=WTA_S], c(mean employed) row col format(%9.1f)*

*table loc2 sex agecategor [aw=WTA_S], c(mean unemploy) row col format(%9.1f)*

*table loc2 sex agecategor [aw=WTA_S], c(mean notactive) row col format(%9.1f)*

Table 5.5

*gen s4aq20urb=s4aq20 if loc2==1*

*gen s4aq20rur=s4aq20 if loc2==2*

*tabout s4aq20urb s4aq20rur s4aq20 sex using Table_5.5.xls [aw=WTA_S] ///*

*if age>14, replace c(col)*

Table 5.14

*tabout s4aq24 s4aq25 s4aq19 s4aq26 s4aq27 s4aq28 s4aq30 s4aq29 sex ///*

*using table_5.14_a.xls [aw=WTA_S] if age>14, c(freq col) replace*

1. **Syntax for Sampled Tables in Chapter Six**

Setting up data

*use "PARTA/SEC5a.dta"*

*merge 1:1 HID PID using "PARTA/SEC5b.dta"*

*drop _merge*

*merge 1:1 HID PID using "PARTA/SEC1.dta"*

*drop _m*

*merge m:1 HID using "AGGREGATES/00_GHA_BASICINFO.dta"*

*drop _merge*

*Table 6.2*

*replace s5aq2=. if s5aq2==0*

*replace s5aq3=. if s5aq3==0 | s5aq3==3*

*gen re_mig=1 if s5aq2==1*

*replace re_mig=1 if s5aq3==1 & s5aq1==1*

*replace re_mig=2 if s5aq1==2 & s5aq2==2 & re_mig==.*

*replace re_mig=3 if s5aq1==1 & re_mig==.*

*tab re_mig [aw=WTA_S] if s1q5y>6*

*recode re_mig (2=1 "In-Migrants") (1=2 "Return Migrants") ///*

*(3=3 "Non Migrants"), gen (mig_status)*

*tabout region mig_status if s1q5y>6 [aw=WTA_S] using table_6_2.xls, ///*

*c(row) f(1) npos(row) nlab(Sample size) layout(cb) replace*

Table 6.7

*tabout s5aq10 loc5 if s1q5y>6 [aw=WTA_S] using table_6_7.xls, ///*

*c(col) f(1) npos(row) nlab(Sample size) layout(cb) replace*

Table 6.13a

*replace s5bq13a=. if s5bq13a==71 | s5bq13a==0*

*replace s5bq13b=. if s5bq13b>=47*

*tab s5bq13a s1q2 [aw=WTA_S], col fre*

*tab s5bq13b s1q2 [aw=WTA_S], col fre*

1. **Syntax for Sampled Tables in Chapter Seven**

Setting up data

*global path1="/Users/samuelannim/Documents/Ghana Datasets/GLSS/GLSS/GLSS 6/GLSS6_Stata/"*

*global path2 = "/Users/samuelannim/Documents/Ghana Datasets/GLSS/GLSS/GLSS 6/GLSS6_Stata/PARTA/"*

*use "$path2/SEC7.dta"*

*merge 1:1 HID using "$path1/AGGREGATES/00_GHA_BASICINFO.dta"*

*gen urbanAreas=loc5 if loc5<3*

*gen ruralAreas=loc5 if loc5>2*

*lab val urbanA loc5*

*lab val ruralA loc5*

Table 7.1

gen s7aq1_urb=s7aq1 if loc2==1

gen s7aq1_rur=s7aq1 if loc2==2

tabout s7aq1* loc5 [aw=WTA_S] using table_7.1.xls, c(col) f(1) ///

nlab(Sample size) layout(cb) replace

Table 7.6

g*en s7fq1_urb=s7fq1 if loc2==1*

*lab val s7fq1_urb s7fq1*

*gen s7fq1_rur=s7fq1 if loc2==2*

*lab val s7fq1_rur s7fq1*

*tabout s7fq1* loc5 [aw=WTA_S] using table_7.6.xls, c(col) f(1) ///*

*nlab(Sample size) layout(cb) replace*

1. **Syntax for Sampled Tables in Chapter Eight**

Setting up data

*global path1="/Users/samuelannim/Documents/Ghana Datasets/GLSS/GLSS/GLSS /// 6/GLSS6_Stata/"*

*global path2 = "/Users/samuelannim/Documents/Ghana Datasets/GLSS/GLSS/GLSS /// 6/GLSS6_Stata/PARTA"*

*global path3 = "/Users/samuelannim/Documents/Ghana Datasets/GLSS/GLSS/GLSS /// 6/GLSS6_Stata/PARTB"*

*global path4 ="/Users/samuelannim/Documents/Ghana Datasets/GLSS/GLSS/GLSS /// 6/GLSS6_Stata/SECTION 10"*

*use "$path3/sec8a2", clear*

*rename hid HID*

*merge m:1 HID using "AGGREGATES/00_GHA_BASICINFO.dta"*

*drop if animcd> 13*

*gen own=1 if s8aq20==1*

*gen number = s8aq21a*

*gen value= s8aq21a*s8aq22*

*gen sales= s8aq25*

*gen purchase =s8aq28*

Table 8.2

*tabstat own number value sales purchase [aw= WTA_S ], by( animcd ) stat(sum)*

Table 8.5b

*use "$path3/sec8a2", clear*

*rename hid HID*

*merge m:1 HID using "AGGREGATES/00_GHA_BASICINFO.dta"*

*gen fishFarmers= s8aq20==1*

*gen value= s8aq21a*s8aq22*

*gen purchases= s8aq28*

*gen sales=s8aq25*

*replace value=value/1000000*

*replace sales=sales/1000000*

*replace purch=purch/1000000*

*tabstat fishFarmers value sales purchases [aw= WTA_S] if animcd ==15, by( loc5 ) /// stat(sum) format(%9.1g)*

1. **Syntax for Sampled Tables in Chapter Nine**

Setting up data

*global path1="/Users/samuelannim/Documents/Ghana Datasets/GLSS/GLSS/GLSS 6/GLSS6_Stata/"*

*global path2 = "/Users/samuelannim/Documents/Ghana Datasets/GLSS/GLSS/GLSS 6/GLSS6_Stata/PARTA"*

*global path3 ="/Users/samuelannim/Documents/Ghana Datasets/GLSS/GLSS/GLSS 6/GLSS6_Stata/SECTION 10"*

*global path4="/Users/samuelannim/Documents/Ghana Datasets/GLSS/GLSS/GLSS 6/GLSS6_Stata/AGGREGATES"*

*use "$path2/sec6", clear*

*merge m:1 HID using "$path4/00_GHA_BASICINFO.dta"*

*keep region clust HID s6q1 s6q9 COUNTRY- loc2*

*merge 1:m HID using "$path3/sec10A"*

*drop _merge*

**rename PID pid*

**rename s10aq3 PID*

*merge m:1 HID PID using "$path2/sec1"*

**drop if _mer==2*

*drop _merge*

*recode s10aq5 (1010/3320=1 Manufacturing)(4510/4799=2 Trading)(111/990 3510/4390 4911/9900=3 Others), gen(charcterNonfarm)*

Table 9.2

*drop if s10aq13==98*

*tab s10aq13 s1q2 [aw= WTA_S ] if charcterNonfarm==1, col nofreq*

*tab s10aq13 s1q2 [aw= WTA_S ] if charcterNonfarm==2, col nofreq*

*tab s10aq13 s1q2 [aw= WTA_S ] if charcterNonfarm==3, col nofreq*

*tab s10aq13 charcterNonfarm [aw= WTA_S ] , col nofreq*

*Table 9.5*

*drop if entno==.*

*bys HID PID: gen numberEnter=_N*

*keep HID PID WTA_S s1q2 numberEnter charcterNonfarm*

*bys HID PID: keep if _n==1*

*merge 1:m HID PID using "$path1/expenditure"*

*keep if _merge==3*

*replace amount12months= amount12months/ numberEnter*

*replace amount3months= amount3months/ numberEnter*

*replace amount2months= amount2months/ numberEnter*

*table s10f2n charcterNonfarm [aw = WTA_S ], content(mean amount12months ) format(%12.1f) col row*

1. **Syntax for Sampled Tables in Chapter Ten**

Setting up data

*use 00_GHA_BASICINFO.dta,clear*

*merge 1:1 HID using 01_GHA_EXPFOOD.dta, gen(merge1)*

*merge 1:1 HID using 02_GHA_EXPALCH.dta, gen(merge2)*

*merge 1:1 HID using 03_GHA_EXPCLTH.dta, gen(merge3)*

*merge 1:1 HID using 04_GHA_EXPHOUS.dta, gen(merge4)*

*merge 1:1 HID using 05_GHA_EXPFURN.dta, gen(merge5)*

*merge 1:1 HID using 06_GHA_EXPHLTH.dta, gen(merge6)*

*merge 1:1 HID using 07_GHA_EXPTRSP.dta, gen(merge7)*

*merge 1:1 HID using 08_GHA_EXPCMNQ.dta, gen(merge8)*

*merge 1:1 HID using 09_GHA_EXPRCRE.dta, gen(merge9)*

*merge 1:1 HID using 10_GHA_EXPEDUC.dta, gen(merge10)*

*merge 1:1 HID using 11_GHA_EXPHOTL.dta, gen(merge11)*

*merge 1:1 HID using 12_GHA_EXPMISC.dta, gen(merge12)*

*merge 1:1 HID using POV_GH_2013.dta, gen(merge13)*

*save hsehldxpenditure,replace*

*use hsehldxpenditure.dta, clear*

*Table 10.4.*

*egen Food_Act_Exp=rowtotal(FD_B FDNONALC_B HOTCAT_RS)*

*sum Food_Act_Exp [aw=WTA_S]*

*egen Food_impu_Exp=rowtotal(FD_P FDNONALC_P)*

*sum Food_impu_Exp[aw= WTA_S]*

*gen tot_food_exp=(Food_Act_Exp+Food_impu_Exp)*

*sum tot_food_exp[aw= WTA_S]*

**Total Housing Expenditure*

**Housing_Exp including rental*

*egen Hou_Act_Exp=rowtotal ///*

*(HSREPAIR HSWATER HSFUEL HSKEROSENE HSDIESEL HSUTILITY RENTALL)*

*sum Hou_Act_Exp[aw= WTA_S]*

**Calculating other expenditure*

**food and housing*

*egen foodhousing=rowtotal(Hou_Act_Exp tot_food_exp)*

*sum foodhousing[aw= WTA_S]*

**other expenditure*

*gen otherexpenditure=HHEXP_NR-foodhousing*

*sum otherexpenditure[aw= WTA_S]*

****COLUMN TWO******

**Per Capita Income for total expenditure*

*gen Per_Capita_Income=HHEXP_NR/hhsize*

*sum Per_Capita_Income[aw= WTA_S]*

**Per capita food expenditure*

*gen Pcefoodactual=Food_Act_Exp/hhsize*

*sum Pcefoodactual[aw=weight]*

*gen Pcefoodimputed=Food_impu_Exp/hhsize*

*sum Pcefoodimputed[aw= WTA_S]*

*gen Pcefoodhousing=Hou_Act_Exp/hhsize*

*sum Pcefoodhousing[aw= WTA_S]*

*gen Pceotherexp=otherexpenditure/hhsize*

*sum Pceotherexp[aw= WTA_S]*

**Third Column: Estimated total annual expenditure*

*gen foodactualtotest=3668.14*6.602*

*display foodactualtotest*

*gen foodimputedtotest=675.55*6.602*

*display foodimputedtotest*

*gen housingtotest=1154.27*6.602*

*display housingtotest*

*gen othertotest=3817.701*6.602*

*display othertotest*

**Last Column: Percentage share of total expenditure*

*gen foodactualpershare=24217.06/61502.00*100*

*display foodactualpershare*

*gen foodimputedpershare=4459.98/61502.00*100*

*display foodimputedpershare*

*gen housingpershare=7620.4907/61502.00*100*

*display housingpershare*

*gen otherexppershare=25204.463/61502.00*100*

*display otherexppershare*

Table 10.16

tab s7aq1 loc5[aw=WTA_S] ,sum(HHEXP_NR) nost nof noobs

Table 10.17

tab OWNTYPE loc5[aw=WTA_S],sum(HHEXP_NR) nost nof noobstab

1. **Syntax for Sampled Tables in Chapter Eleven**

Setting up data

*use "PARTA/SEC1.dta"*

*renames HID PID \ hid pid*

*use "PARTB/sec12a.dta", clear*

**isid hid pid*

*drop if pid==.*

*save "PARTB/sec12a_v1.dta", replace*

*use "PARTA/SEC1.dta"*

*renames HID PID \ hid pid*

*merge 1:1 hid pid using "PARTB/sec12a_v1.dta"*

*drop _merge*

*save "PARTB/sec12a_v1.dta", replace*

*use "PARTB/sec12c.dta", clear*

**isid hid pid*

*drop if pid==.*

*duplicates tag hid pid, gen(dup)*

*duplicates drop hid pid, force*

*save "PARTB/sec12c_v1.dta", replace*

*use "PARTB/sec12a_v1.dta", clear*

*merge 1:1 hid pid using "PARTB/sec12c_v1.dta"*

*drop _merge*

*rename hid HID*

*merge m:1 HID using "AGGREGATES/00_GHA_BASICINFO.dta"*

Table 11.2

*tab s12aq4a s1q2 [aw=WTA_S], row*

*gen s12aq4a_ag= s12aq4a if loc5==1*

*gen s12aq4a_ou= s12aq4a if loc5==2*

*gen s12aq4a_rc= s12aq4a if loc5==3*

*gen s12aq4a_rf= s12aq4a if loc5==4*

*gen s12aq4a_rs= s12aq4a if loc5==5*

*gen s12aq4b_ag= s12aq4b if loc5==1*

*gen s12aq4b_ou= s12aq4b if loc5==2*

*gen s12aq4b_rc= s12aq4b if loc5==3*

*gen s12aq4b_rf= s12aq4b if loc5==4*

*gen s12aq4b_rs= s12aq4b if loc5==5*

*gen s12aq4c_ag= s12aq4c if loc5==1*

*gen s12aq4c_ou= s12aq4c if loc5==2*

*gen s12aq4c_rc= s12aq4c if loc5==3*

*gen s12aq4c_rf= s12aq4c if loc5==4*

*gen s12aq4c_rs= s12aq4c if loc5==5*

*gen s12aq4d_ag= s12aq4d if loc5==1*

*gen s12aq4d_ou= s12aq4d if loc5==2*

*gen s12aq4d_rc= s12aq4d if loc5==3*

*gen s12aq4d_rf= s12aq4d if loc5==4*

*gen s12aq4d_rs= s12aq4d if loc5==5*

*gen s12aq4e_ag= s12aq4e if loc5==1*

*gen s12aq4e_ou= s12aq4e if loc5==2*

*gen s12aq4e_rc= s12aq4e if loc5==3*

*gen s12aq4e_rf= s12aq4e if loc5==4*

*gen s12aq4e_rs= s12aq4e if loc5==5*

*gen s12aq4f_ag= s12aq4f if loc5==1*

*gen s12aq4f_ou= s12aq4f if loc5==2*

*gen s12aq4f_rc= s12aq4f if loc5==3*

*gen s12aq4f_rf= s12aq4f if loc5==4*

*gen s12aq4f_rs= s12aq4f if loc5==5*

*tabout s12aq4a_* s12aq4a s12aq4b_* s12aq4b s12aq4c_* s12aq4c ///*

*s12aq4d_* s12aq4d s12aq4e_* s12aq4e s12aq4f_* s12aq4f s1q2 ///*

*[aw=WTA_S] using table_11.2.xls, c(row) f(1) nlab(Sample size) layout(cb) replace*

Table 11.3:

*recode s12aq16 (1=1 yes) (2=0 no), gen(insurance)*

*bys HID: egen insurance_hh=sum(insurance)*

*replace insurance_hh=1 if insurance_hh>1*

*tab loc5 insurance_hh [aw=WTA_S] if s1q3==1, r*

*tab loc2 insurance_hh [aw=WTA_S] if s1q3==1, r*

1. **Syntax for Sampled Tables in Chapter Twelve**

Setting up data

*use gps-sec0.dta*

*merge 1:1 clust nh pid using gps-sec13a.dta*

*drop _merge*

*merge 1:1 clust nh pid using gps-sec13b.dta*

*drop _merge*

*merge 1:1 clust nh pid using gps-sec13c.dta*

*drop _merge*

*merge 1:1 clust nh pid using gps-sec13d.dta*

*drop _merge*

*merge 1:1 clust nh pid using gps-sec13e.dta*

*drop _merge*

*merge 1:1 clust nh pid using gps-sec13f.dta*

*drop _merge*

*merge 1:1 clust nh pid using gps-sec13g.dta*

*drop _merge*

*saveold gps_merge.dta, replace*

Table 12.9

*tabout s13bq7 region [aw=WTA_S] using table_12_9.xls, ///*

*c(col) f(1) npos(row) nlab(Sample size) layout(cb) replace*

Table 12.13

*tabout s13cq18a s13cq18b region [aw=WTA_S] using table_12_13.xls, ///*

*c(col) f(1) npos(row) nlab(Sample size) layout(cb) replace*

Table 12.22

*replace s13gq4=. if s13gq4==7*

*tabout s13gq4 region [aw=WTA_S] using table_12_22.xls, ///*

*c(col) f(1) npos(row) nlab(Sample size) layout(cb) replace*

Syntax for generating educational attainment using the GDHS

*use "/Users/samuelannim/Documents/Ghana /// Datasets/GDHS/GH_2014_DHS/GHPR70FL.DTA"*

*recode hv109 (0=0 "no education") (1 2 = 1 "Less MSLC/BECE") (3=2 "MSLC/BECE") ///*

*(4 5=4 "Secondary and Higher") (8=.), gen(educ_comp)*

*tab educ_comp [aw= hv005] if hv105>14*
